# Electrolysis in Dermatology

**Published:** 1914-12

**Authors:** Joseph Spangenthal

**Affiliations:** Dermatologist to the Good Samaritan Dispensary and Emergency Hospital, Buffalo, N. Y.; 595 Lafayette Ave


### Electrolysis in Dermatology.

By JOSEPH SPANGENTHAL, M. D.

Dermatologist to the Good Samaritan Dispensary and Emergency Hospital, Buffalo, N. Y.

The employment of the electric needle is of the utmost importance to the Dermatologist. By this method of treatment, most of the minor operations of the skin are performed in a bloodless and painless manner.

The paraphernalia required are a galvanic battery consisting of 30 to 40 cells, a rheostat to control the strength of the current, a milliampere-meter to measure the amount of current, a large flat sponge electrode, and a needle holder with variously shaped needles.

The sponge electrode is attached to the positive pole, and the needle holder to the negative. The attachment of the needle holder to the negative pole is essential for the following reasons:

Electrolytic action is a chemical one, in which the current at the negative pole has an alkaline caustic effect, producing liquefaction and disintegration of tissue, resulting in a soft and pliable cicatrix. The current at the positive pole has an acid caustic effect, producing a cicatrix hard and unyielding.

Moreover, by the use of a steel needle at the positive pole, there would be the objectionable deposit of iron rust in the tissue due to oxidation.

The diseases and abnormalities of the skin which are removed advantageously by the employment of electrolysis, are:

1. Superfluous Hair.
2. Verrucae and Moles.
3. Naevi.
4. Epitheliomata.
5. Rosacia and Telangiectases.

**Superfluous Hair.**—For the permanent removal of superfluous hair, occurring on women's faces, and for which relief is so urgently sought electrolysis has its greatest value. The operation if properly done, is permanently effectual. As each hair is treated individually, only those cases should be selected, in which the hairs are coarse and not too numerous.

The parts to be operated upon, having been cleansed with alcohol, the patient places one hand on a large sponge electrode, saturated with salt solution. The operator fastens the needle holder to the negative pole. The needle should be straight, having a bulbous extremity. The needle is carefully introduced into the hair follicle, following the direction of the hair shaft. The current must not be over 1 milliampere. Hydrogen froth will almost immediately be seen issuing from the follicle.

After a variable time of 10 to 20 seconds, the follicle will have been destroyed, and if properly done, the hair should be lifted from its sheath without any traction. A skillful operator will be able to remove about 75 hairs within an hour, with a regrowth of 10%. These may be subsequently removed.

**Warts and Moles.**—When we consider the frequency with which these benign growths undergo malignant degeneration, and how easily they may be removed by electrolysis, the advisability of their early destruction becomes apparent. The principle underlying their destruction, is to cut off their source of nourishment by destroying their blood supply. To make the operation painless, inject a few drops of a local anaesthetic just beneath its base. The patient placing one hand upon the electrode, an ordinary small, half-curved, surgeon's needle is transfixed underneath the wart or mole, on a line with the skin. A current of 2 to 5 milliamperes is employed. There will be a blanching of the growth due to the destruction of its blood vessels. After a sufficient length of time, depending upon the strength of the current and the size of the growth, destruction will be completed.

**Naevi.**—Under this classification are the many varieties of birthmarks such as, Naevus Vascularis, Pigmentosus, Linearis, etc.

These are all amenable to electrolytic destruction. The technique is the same as that employed for the removal of moles. Where the growth is very large, the operation must be repeated several times at intervals.

**Epitheliomata.**—Electrolysis has its place in the destruction of certain varieties of epitheliomata. In warts and moles that are in the incipiency of malignant degeneration, their undermining by means of the electric needle, is all that may be necessary for their eradication. This method of treatment also applies to the superficial variety. Should the lesion have advanced to the stage of ulceration, electrolysis should be employed preliminary to subsequent treatment.

**Rosacia and Telangiectasis.**—The treatment of the dilated capillaries as observed in these diseases of the skin, is operative; the best method consisting in their destruction by means of the electric needle.

A fine half-curved needle is inserted into each dilated vessel, and within a few seconds destruction is completed.

Other diseases of the skin which may suggest themselves to which electrolysis is applicable are, Lupus Vulgaris, Xanthoma, Molluscum Contagiosum, and small Fibromata.

Considering the ease with which the above named diseases and abnormalities are operated upon without hemorrhage and in most cases without pain, the Dermatologist values the electric needle as one of the most important instruments in his possession for the surgical treatment of skin affections.

595 Lafayette Ave.

---

**Creatinine as a Test of Renal Function.** Neubauer, "Münch. Med. Woch.," states that the normal daily excretion of creatinine is 0.8 to 2.4 grams. It is produced endogenically and does not vary appreciably with diet. With the patient on his usual diet, a 24 hour specimen of urine is estimated. Having thus determined the individual norm, a dose of 1.50 grams of creatinine is given in 100 c.c. of sweetened water or, if there is gastric disturbance, hypodermatically. A series of 6-hour specimens are collected for a full 24-hour day. The increase in creatinine to be expected is 0.9 to 1.2 grams; 0.1 to 0.45; for the first two periods, 70% to 100% of the administered creatinine being normally eliminated in the first 12 hours. In serious disturbance of the renal function, the increase may be only 0.3 gram and the elimination of the ingested or injected creatinine is disturbed over the four 6-hour periods or may be further delayed. Autenrieth's colorimeter is used. Attention is also called to a through discussion of creatin and creatinine by Victor Caryl Myers of the N. Y. P. G. Medical School and Hospital, in the "Post Graduate" for June, 1914.

---

**Automobile and Railroad Fatalities.** In 1912, 691 persons were killed in 22 American cities of 100,000 and more population. 270 persons were killed by railroads in the whole country. The highest number killed by railroads in any one year was 647 in 1907.—"Lit. Digest." (Note: It seems plausible that automobiles, encountered at every street crossing, should kill many more persons than railroads but the U. S. Census report for 1912 gives the actual number of persons killed by railroads in 1912, in the registration area, as 8,209; and the deaths from automobiles in the same area and for the same year as 1,758. There is no use moralizing on incorrect statements.)

---

**Large Adeno-Osteo-Fibroma of Breast.** de la Volpiliere and Merle, "Arch. Med. du Centre," July, 1914. Woman aged 36, 2 children, has never nursed nor had a traumatism of the breast. Small tumor palpable in left breast at age of 14, increasing to size of orange after first pregnancy at age of 25. Not increased by second pregnancy at age of 27. Tumor has increased intermittently until it has reached the size of an adults' head. Successful excision. Histologic demonstration as above.

